# Selection of Human Cytomegalovirus Mutants with Resistance against PDGFRα-Derived Entry Inhibitors

**DOI:** 10.3390/v13061094

**Published:** 2021-06-08

**Authors:** Kerstin Laib Sampaio, Carolin Lutz, Rebecca Engels, Dagmar Stöhr, Christian Sinzger

**Affiliations:** Institute for Virology, Ulm University Medical Center, 89081 Ulm, Germany; kerstin.laib@uni-ulm.de (K.L.S.); carolin.lutz@uni-ulm.de (C.L.); Engels@mvp.lmu.de (R.E.); dagmar.stoehr@uniklinik-ulm.de (D.S.)

**Keywords:** human cytomegalovirus, entry inhibitor, drug resistance, PDGFRα

## Abstract

The human cytomegalovirus (HCMV) infects fibroblasts via an interaction of its envelope glycoprotein gO with the cellular platelet-derived growth factor receptor alpha (PDGFRα), and soluble derivatives of this receptor can inhibit viral entry. We aimed to select mutants with resistance against PDGFRα-Fc and the PDGFRα-derived peptides GT40 and IK40 to gain insight into the underlying mechanisms and determine the genetic barrier to resistance. An error-prone variant of strain AD169 was propagated in the presence of inhibitors, cell cultures were monitored weekly for signs of increased viral growth, and selected viruses were tested regarding their sensitivity to the inhibitor. Resistant virus was analyzed by DNA sequencing, candidate mutations were transferred into AD169 clone pHB5 by seamless mutagenesis, and reconstituted virus was again tested for loss of sensitivity by dose-response analyses. An S48Y mutation in gO was identified that conferred a three-fold loss of sensitivity against PDGFRα-Fc, a combination of mutations in gO, gH, gB and gN reduced sensitivity to GT40 by factor 4, and no loss of sensitivity occurred with IK40. The resistance-conferring mutations support the notion that PDGFRα-Fc and GT40 perturb the interaction of gO with its receptor, but the relatively weak effect indicates a high genetic barrier to resistance.

## 1. Introduction

Human cytomegalovirus (HCMV) is a ubiquitously distributed member of the herpesvirus family that causes relevant morbidity and mortality under conditions of low immunity, e.g., when infection occurs in transplant recipients or in utero. Although antiviral drugs are available, certain challenges for successful treatment remain. All drugs that are currently approved for treatment target the viral polymerase, and are limited due to adverse effects and development of resistance including cross-resistance [1]. Recently, the viral terminase was defined as an additional target, and the terminase inhibitor letermovir was approved for prophylaxis [2]. While this drug appears to be less toxic, the appearance of resistance-conferring mutations have also been reported [3]. Particularly for the situation of intrauterine infection, there is an ongoing need for alternative non-toxic antiviral strategies to increase the number of treatment options for HCMV infections.

A relatively new field in antiviral research is the inhibition of entry. HIV is the only virus for which entry inhibitors have been approved to date, including both small compound drugs and a peptide [4,5]. For HBV and HDV, a promising peptide-based entry inhibitor is currently being investigated in clinical trials [6]. Entry of HCMV into its target cells is mediated by two envelope complexes containing the glycoproteins gH and gL, the trimer gH/gL/gO and the pentamer gH/gL/pUL128/pUL130/pUL131A, that interact with the cellular receptors PDGFRα and Nrp2, respectively, and are assumed to trigger the fusion protein gB upon this interaction [7,8,9,10,11]. Binding of gO to PDGFRα can be blocked by a soluble derivative of this receptor (PDGFRα-Fc) which effectively inhibits viral entry and may have potential for drug development [9,12].

Since all HCMV strains appear to rely on PDGFRα as a receptor for entry into fibroblasts, PDGFRα-Fc is probably less vulnerable to the development of resistance by the virus than the currently available antivirals, because a reduction in binding to the inhibitor would likely also affect interaction with the receptor on the cell surface. It is therefore conceivable that the genetic barrier to resistance will be high. Two peptides from an overlapping 40mer peptide set covering the extracellular part of PDGFRα (GT40 and IK40) can also inhibit HCMV infection [9] but it is unclear whether they also act by the same mechanism as the full-length protein and whether cross resistance could occur between peptides and PDGFRα-Fc. This study therefore aimed at selection of HCMV mutants with reduced sensitivity to the entry inhibitor PDGFRα-Fc and the PDGFRα-derived inhibitory 40mer peptides GT40 and IK40, to determine the genetic barrier to resistance, and to obtain a hint at the interaction sites between the gH/gL/gO trimer and PDGFRα-derived entry inhibitors.

## 2. Materials and Methods

### 2.1. Cells

For propagation, human foreskin fibroblasts (HFFs) were either grown in minimal essential medium with GlutaMAX (Thermo Fisher Scientific, Waltham, MA, USA), 5% fetal bovine serum (PAN Biotech, Aidenbach, Germany), 100 µg/mL gentamicin (Sigma-Aldrich, St. Louis, MO, USA), and 0.5 ng/mL basic fibroblast growth factor (bFGF; Thermo Fisher Scientific) or Dulbecco’s Modified Eagle Medium with GlutaMAX (Thermo Fisher Scientific) supplemented with 10% fetal bovine serum and 100 µg/mL gentamicin. During infection experiments, bFGF was omitted from the medium to avoid competition of this growth factor with the virus for binding to heparan sulfate proteoglycans on the cell surface. Cell-culture microplates were coated with 0.1% gelatin (Sigma-Aldrich) prior to seeding of cells.

### 2.2. Viruses

For selection of resistance-conferring mutations we used the proofreading-deficient AD169 mutant HGUL54dD413 [13], as this mutant is expected to increase the error rate during replication of the viral genome. For marker transfer experiments, we used the AD169-derived bacterial artificial chromosome (BAC) pHB5 in *E. coli* GS1783 and virus reconstituted thereof by transfection of BAC DNA into HFFs [14]. For preparation of cell-free virus stocks, supernatants from productively infected cultures were centrifuged for 10 min at 2790× *g* to remove cells and debris and stored at −80 °C in aliquots until used in experiments.

### 2.3. PDGFRα-Derived Soluble Receptor and Peptides

Recombinant PDGFRα-Fc fusion protein (R&D, Minneapolis, MN, USA) was dissolved in phosphate buffered saline (PBS) at a concentration of 500 μg/mL. Peptides derived from the extracellular domain of human PDGFRα were obtained from Phtdpeptides (Shanghai, China) at a purity of >95% with the following sequence: GT40: GRHIYIYVPDPDVAFVPLGMTDYLVIVEDDDSAIIPCRTT and IK40: IKVPSIKLVYTLTVPEATVKDSGDYECAARQATREVKEMK. Peptides were initially dissolved in dimethyl sulfoxide (DMSO, Sigma-Aldrich) at a concentration of 25 mmol/L and then diluted dropwise into prewarmed growth medium under vigorous shaking to obtain a stock solution of 250 µmol/L.

### 2.4. Selection of Viruses with Resistance against PDGFRα-Fc or the PDGFRα-Derived Peptides IK40 and GT40

To induce mutations that reduce sensitivity to the soluble receptor PDGFRα-Fc, cells infected with the error-prone RV-HGULdD413 mutant were co-seeded once per week with uninfected HFFs in the presence of 30 ng/mL of the inhibitor, which corresponds to 2 × EC90 (the concentration at which infection with cell-free virus is reduced by 90%). For passage, infection rates of HFFs were estimated by assessing the cytopathic effect by phase contrast microscopy. Cells were then detached with trypsin/EDTA, suspended in medium, counted, and reseeded with an excess of uninfected HFFs in the presence of the inhibitor. The coculture was incubated at 37 °C for 1 week to allow virus production and cell-associated spread. When selection cultures showed enlarged foci, indicating decreased sensitivity to the inhibitor, infectious supernatants were collected after the inhibitors had been omitted from the culture media for several days. Supernatants were then subjected to plaque purification by limiting dilution infections in HFFs on 96-well plates in the presence of inhibitors, similar to the selection conditions described above. Wells that exhibited solitary enlarged foci were selected, and the supernatant was exchanged for inhibitor-free medium to produce infectious supernatant for further propagation.

To induce mutations that reduce sensitivity to the PDGFRα-derived peptides IK40 and GT40, selection experiments were performed essentially identically, with the following modification: peptides were added at 25 µmol/L and were replenished twice per week as they were less stable than the full-length protein.

### 2.5. Dose–Response Curves

For testing the inhibitory effect of PDGFRα-Fc or PDGFRα-Fc derived peptides on HCMV, the inhibitors were serially diluted in medium and mixed with virus preparations at a concentration resulting in a final infection multiplicity of one infectious unit per cell. The mixtures were incubated for 2 h at 37 °C and then added to HFFs that have been seeded on gelatin-coated 96-well plates at a density of 1.5 × 10^4^ cells the day before. Cells were incubated overnight, and infection efficiencies were measured by immunofluorescence detection of viral immediate early proteins as described below. EC50 values were determined by non-linear regression using Sigma Plot software.

### 2.6. Detection of Viral Immediate Early Proteins by Indirect Immunofluorescence

Infected cells were fixed with 80% acetone for 5 min at room temperature, washed with PBS and incubated at 37 °C for at least 90 min with antibody E13 (Argene/Biomerieux, Marcy-l’Étoile, France) directed against viral immediate early (IE) antigens (UL122/123) or MAb 63-27 directed against pUL123 (kindly provided by W. Britt). Cells were washed with PBS, incubated for 60 min with Cy3-goat-anti-mouse Ig F(ab’)2 fragments (Jackson ImmunoResearch, West Grove, PA, USA), washed with PBS and counterstained with 4′,6-diamidino-2-phenylindole (DAPI, Sigma-Aldrich). Blocking of potential non-specific binding was not necessary with this set of antibodies. This procedure resulted in nuclear red fluorescence of infected cells and nuclear blue fluorescence of all cells. Images were acquired by fluorescence microscopy using an Axio Observer D1 microscope (Zeiss, Oberkochen, Germany), quantification was performed using Zen software (Zeiss), and infection efficiency was calculated as the ratio of the number of red and blue nuclei.

### 2.7. Generation of Mutant Viruses

Mutations were introduced into viral genomes that were available as BACs using the markerless mutagenesis protocol developed by Tischer et al. [15].

For introduction of candidate resistance-conferring mutations into AD169-derived BAC pHB5, plasmid pEP-Kan-S served as a template to generate recombination fragments that consisted of the 18-bp I-Sce I restriction site and a kanamycin resistance cassette flanked by repeated sequences containing homology to the desired site of insertion in the HCMV genome. A two-step PCR was performed with a long forward primer and a short universal kanamycin reverse primer in a first round of amplification (Table 1). After purification of products by NucleoSpin PCR Clean-up Kit (Macherey Nagel, Düren, Germany), a second amplification was conducted with the respective short forward and long reverse primers (Table 1) to obtain the final recombination fragment, which was then inserted by electroporation into recombination-activated GS1783 harboring the pHB5 genome. After selection of kanamycin-resistant clones, all non-HCMV sequences were removed by intra-bacterial I-Sce I digestion and a subsequent red recombination step. For each mutant, two independent Kanamycin-sensitive clones were selected, and BAC-DNA was isolated using the NucleoBond Xtra Midi Kit (Macherey-Nagel). Each mutant was analyzed by RFLA, and Sanger sequencing and virus was reconstituted by transfection of the purified BAC-DNA into HFFs with the K2 Transfection System (Biontex Laboratories, München, Germany).

For generation of dual-labeled HCMV strain TB40-BAC-KL7-SE-UL32EGFP-UL100mCherry, the EGFP template cassette was amplified in a first step from plasmid pEP-EGFPin (Addgene plasmid #60961) with primers EGFP for and EGFP rev (Table 2) and inserted into TB40-BAC-KL7-SE [16] by seamless mutagenesis as described above. After removal of the selection marker, an mCherry-containing recombination fragment was generated from plasmid pEP-mCherry-in [17] with primers mCherry for and mCherry rev (Table 2) and introduced into the genome by a second round of mutagenesis. The final TB40-BAC-KL7-SE-UL32EGFP-UL100mCherry genome was verified by RFLA and sequencing, and infectious virus was generated by transfection into HFFs as described above.

### 2.8. Retransformation of E. coli with Viral Genomes and Analysis of the DNA Sequence

Viral DNA of the plaque-purified GT40-selectant was prepared from infected HFFs at 4 d p.i. according to the protocol of Hirt [18], 5 µg DNA were electroporated into 75 µL MegaX DH10B electrocompetent cells (Thermo Fisher Scientific) and grown on LB-agar plates containing 25 µg/mL chloramphenicol. DNA from cultures of four individual clones was purified with NucleoBond Xtra Midi Kit and transfected back into HFFs by lipofection (K2 Transfection System). Infectious supernatants from three individual clones could be harvested and subsequently analyzed in dose-response curves. Hirt-DNA from clone 1 was obtained as described above and subjected to whole genome sequencing (SEQ-IT GmbH u. Co. KG, Kaiserslautern, Germany), with indexed libraries generated by the Illumina TruSeq DNA PCR-free Kit and subsequently sequenced on an Illumina MiSeq instrument. The resulting reads were processed with the Geneious Prime software (Biomatters, Auckland, New Zealand) and compared to the published genome of the pHB5-derivate BAC HB15 (GenBank KU317610.1). The complete sequence of clone 1 (HGUL54dD413 selGT40 clone1) is available at the NCBI GenBank with accession number MZ327301.

### 2.9. Quantification of Adsorption and Penetration

HFFs were seeded on gelatin-coated 8-well μ-slide (Ibidi) at a density of 4 × 10^4^ cells per well. Freshly produced cell-free infectious supernatant of TB40-BAC-KL7-SE-UL32EGFP-UL100mCherry was mixed with equal volumes of growth medium containing 60 µmol/L GT40, IK40 or 0.24% DMSO (untreated control) and incubated for 2 h at 37 °C. Medium on cells was exchanged against virus mixtures and incubated for 20 min at 37 °C. Virus mixtures were exchanged against growth medium and cultures were incubated for another 100 min at 37 °C. Cells were fixed with 4% paraformaldehyde for 30 min, washed with PBS, permeabilized for 10 min with PBS containing 1% fetal bovine serum (PAN Biotech), 10% sucrose (Sigma-Aldrich) and 0.5% Nonidet P40 (Sigma-Aldrich), and nuclei were then stained with DAPI (Sigma-Aldrich). Slides were then analyzed by fluorescence microscopy with an Axio Observer D1 microscope (Zeiss). Pictures of each individual channel (native red and green fluorescent signals and blue nuclei) were taken, and particles of 20 cells per condition were counted. The number of all pUL32-EGFP-positive signals per cell was counted as a readout for “adsorption”. The percentage of particles per cell that show only green fluorescence without red fluorescence was determined as a readout for “penetration”.

### 2.10. Statistical Analyses

Datasets with more than two groups of data were analyzed by one-way-ANOVA using the build-in data analyses function of Excel to test whether there are significant differences between the various conditions. If ANOVA indicated significant differences between groups within the data set, appropriate post-hoc analyses (unpaired *t*-tests) were performed to identify groups that differ from the untreated control. Differences between conditions were considered marginally significant when *p*-values were <0.05, significant when *p* values were <0.01 and highly significant when *p* values were <0.001.

## 3. Results

### 3.1. Selection of an AD169 Mutant with Decreased Sensitivity to the Entry Inhibitor PDGFRα-Fc

**Figure 1:**
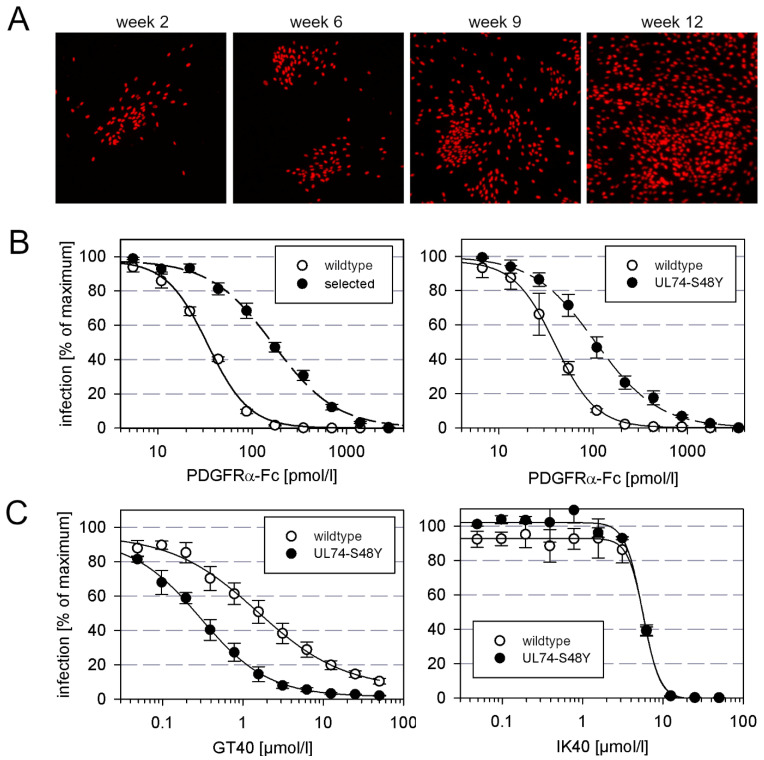
Selection of an AD169 mutant with decreased sensitivity to PDGFRα-Fc. (**A**) Phenotypic change under selection with PDGFRα-Fc. HFFs in T25 culture flasks were infected with the error-prone AD169 mutant HGUL54dD413 and grown in the presence of 2 × EC90 PDGFRα-Fc. After one week, the infected cells were cocultured with fresh uninfected HFFs and the inhibitor PDGFRα-Fc was replenished. This passaging procedure was repeated weekly, and the focus size was visualized in parallel cultures of each passage after 7 d by fixation and immunofluorescence detection of viral immediate early antigen. (**B**) Dose-response analyses of the resistant virus selected with PDGFRα-Fc and the recombinant virus with transfer of the S48Y mutation into UL74, each in comparison with the respective wildtype virus. Virus was pretreated with increasing concentrations of PDGFRα-Fc for 2 h and incubated with HFFs overnight. Cell cultures were then fixed and stained for viral immediate early antigens by immunofluorescence. The fraction of infected cells as compared to untreated controls (maximum) is plotted against the concentration of the inhibitor. Error bars represent the standard error of the mean (SEM). (**C**) Virus preparations of wildtype virus AD169-pHB5 or the UL74-S48Y mutant were pretreated for 2 h with PDGFRα-derived peptides GT40 or IK40 at indicated concentrations and then added to fibroblasts (HFFs). One day after infection, cells were fixed and stained for viral IE antigens by indirect immuno-fluorescence. The fraction of infected cells as compared to untreated controls (maximum) is plotted against the concentration of the inhibitor. Error bars represent the standard error of the mean (SEM).

To test whether HCMV can escape from inhibition by PDGFRα-Fc via the development of resistance-mediating mutations, we tried to select such mutants by continued propagation of the virus in the presence of submaximal inhibitory concentrations of this entry inhibitor. With this approach, we aimed to gain information about the genetic barrier to resistance and identify sites within the trimer that contribute to interaction with its cellular receptor. To accelerate the formation of mutations, we used AD169-HGUL54dD413, a proofreading-deficient UL54-ΔD413 variant of HCMV strain AD169-pHB5 [13]. HFFs infected with this virus were passaged by coculture with uninfected HFFs in the presence of 30 ng/mL PDGFR-alpha-Fc (corresponding to 2 × EC90). At this concentration, cell-free virus would be strongly inhibited, but cell-associated transmission would still allow some degree of viral spread. Mutations that allow cell-free spread in the presence of this entry inhibitor would give the mutant virus a growth advantage, and therefore such mutations should be selected over time. To detect such events, we monitored the cell cultures by visualizing the number and distribution of infected cells. At week 9, the growth pattern changed, indicating cell-free spread (Figure 1A), and from the corresponding supernatant, we isolated a virus that showed a five-fold reduced sensitivity to PDGFRα-Fc as compared to wildtype virus (Figure 1B, left graph). Targeted sequence analyses revealed that gH and gL were unaltered, but in UL74, which encodes for gO, we found a C to A point mutation that translates into a serine to tyrosine mutation at position 48 of the amino acid sequence.

### 3.2. Marker Transfer Confirms That the S48Y Mutation in the N-Terminus of gO Confers Partial Resistance against PDGFRα-Fc

To test, whether the S48Y mutation in gO was responsible for the decreased sensitivity against PDGFRα-Fc, it was introduced into the genetic background of strain AD169 via seamless mutagenesis of the BAC-cloned virus genome pHB5. For this marker transfer experiment, a virus with an intact polymerase gene was chosen to avoid unwanted second site mutations. The mutated virus was reconstituted and analyzed regarding sensitivity to PDGFRα-Fc by dose–response curves in comparison with the parental wildtype virus. These analyses showed that the mutation confers moderate, approximately three-fold, resistance (Figure 1B right graph).

Like the complete extracellular domain of PDGFRα, certain peptide derivatives thereof can also inhibit HCMV infection, albeit at much higher molar concentrations. We were curious about whether the mutation that reduced sensitivity to the complete molecule would also confer resistance against these inhibitory peptides. To address this question, mutant and wildtype virus were compared in dose–response experiments with the PDGFRα-derived 40mer peptides IK40 and GT40. However, in contrast to the previous experiment with the full-length molecule, the S48Y mutation in gO had no effect on sensitivity to IK40 and, surprisingly, even sensitized the virus for inhibition by GT40 (Figure 1C).

### 3.3. The PDGFRα-Derived Peptide GT40 Resembles Full-Length PDGFRα-Fc Regarding the Mode of Action

**Figure 2:**
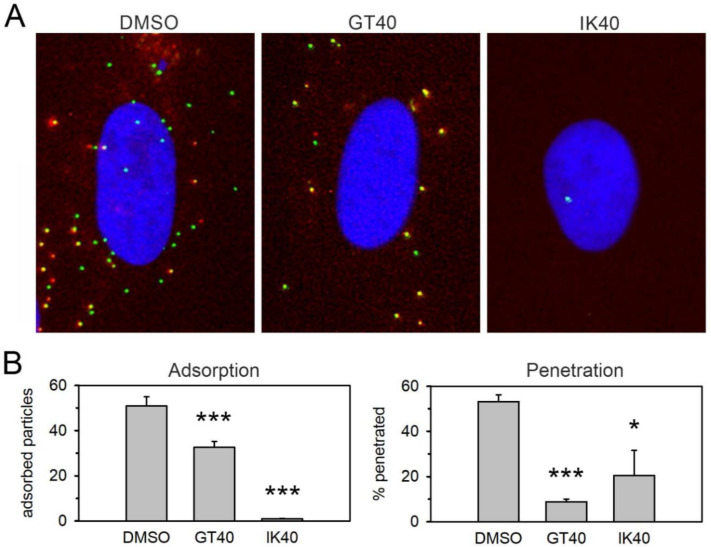
Effect of peptides on adsorption and penetration. A freshly produced virus preparation of TB40-BAC-KL7-SE- UL32EGFP-UL100mCherry was incubated with the respective peptide at a final concentration of 30 µmol/L or a DMSO control at 37 °C for 2 h. Fibroblasts (HFFs) were then infected with the virus–peptide mixture for 20 min at 37 °C, washed with medium, and fixed with paraformaldehyde after a total duration of 2 h. One representative of three experiments is shown. (**A**) Example images showing the effect of the respective peptide as compared to the DMSO control. Enveloped virus particles appear yellow by the superposition of red (envelope; pUL100-mCherry) and green (capsid; pUL32-EGFP) signal. Penetrated particles fluoresce exclusively in green due to loss of the envelope. (**B**) Particle signals from 20 cells were counted for each experimental condition. Adsorption: number of all pUL32-EGFP positive signals per cell. Penetration: percentage of particles per cell that show only green fluorescence without red fluorescence. Bars indicate mean values and error bars indicate the standard error of the mean. Asterisks indicate statistical significance of the differences (* = *p* < 0.05; *** = *p* < 0.001).

This striking discrepancy between PDGFRα-Fc and the PDGFRα-derived peptides concerning the effect of the S48Y mutation in gO suggested that they differ regarding their mode of action and their interaction sites on viral surface proteins. Therefore, we tested whether the PDGFRα peptides also inhibit viral entry in a similar way as previously observed for PDGFRα-Fc [9]. To discriminate whether the peptides act on adsorption, penetration, or both steps, we used a fluorescent HCMV mutant in which the capsid-associated tegument protein pp150 (UL32) is labeled with the green fluorescent protein EGFP and the abundant envelope protein gM (UL100) is labeled with the red fluorescent protein mCherry. This allowed us to count the total number of bound virus particles as a measure of adsorption (all green signals) and to determine the proportion of penetrated virions among those that had bound (green signals without red fluorescence). These analyses showed that the effect of IK40 is almost exclusively due to inhibition of adsorption, whereas for GT40 a large part of the effect is due to inhibition of the penetration step (Figure 2), similar to PDGFRα-Fc [9].

### 3.4. Selection of a Mutant with Decreased Sensitivity to the PDGFRα-Derived Peptide GT40

**Figure 3:**
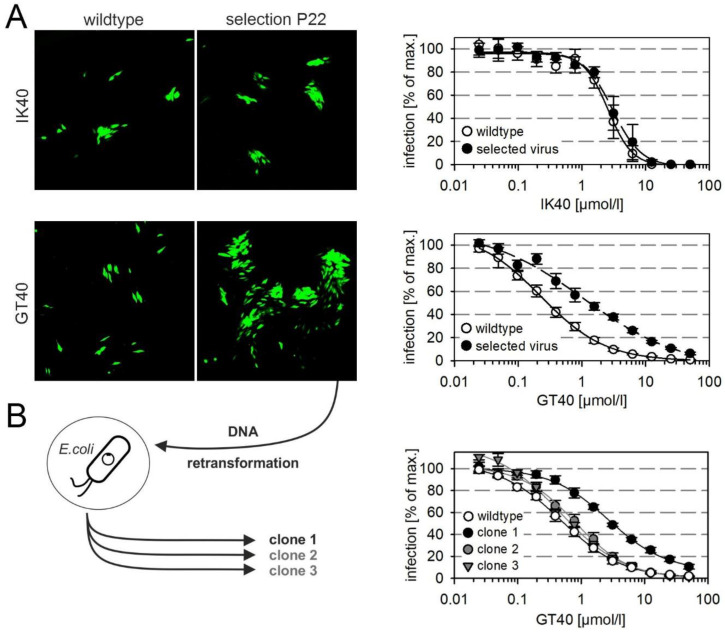
Selection of viruses with resistance against PDGFRα-derived inhibitory peptides. The proofreading-deficient virus RV-HGUL54dD413 (AD169), which expresses the green fluorescent protein under the HCMV-IE promoter, was cultured on HFFs with peptides IK40 and GT40 at submaximal inhibitory dose (25 µmol/L) for a period of 22 passages. Upon selection under GT40, enlarged foci were observed from passage 18. (**A**) Microscopic fluorescence images show the focal growth of wildtype and selected virus as reflected by EGFP expression, and the corresponding dose–response curves show the inhibitory effect of the respective peptide on wildtype and selected virus on cell-free infection of HFFs (n = 6; error bars = standard error of the mean). (**B**) To isolate genetically homogenous clones from the GT40-selectant, viral DNA was prepared from infected cell cultures and introduced into *E. coli* by electroporation. From bacteria with bacterial artificial chromosomes (BACs) containing intact viral genomes, DNA was prepared and transfected into fibroblasts to reconstitute virus. Viruses were then analyzed for sensitivity to the inhibitory peptide GT40 by dose–response experiments (n = 4; error bars = standard error of the mean). One of three clones showed reduced susceptibility to GT40, while the other two clones were almost indistinguishable from wildtype virus.

We next sought to gain further insight into the molecular interactions by the selection of resistance-mediating mutations and therefore propagated the proofreading-deficient HCMV strain AD169-HGUL54dD413, again under selection pressure at conditions of submaximal inhibition, this time using the PDGFRα-derived peptides GT40 and IK40. We were able to select viruses that showed five-fold reduced sensitivity to the peptide GT40 whereas no resistance could be generated against IK40 within the observation period (Figure 3A). To purify individual clones from the selected partially GT40-resistant virus we retransformed a suitable *E. coli* strain with the viral DNA and selected four individual clones that were resistant against chloramphenicol and hence contained HCMV-DNA. Three of the four clones could be reconstituted by transfection into HFFs, and the harvested virus preparations were then analyzed in dose-response curves regarding their sensitivity to GT40. One clone showed six-fold reduced sensitivity and was hence chosen for further analyses (Figure 3B, clone 1).

**Figure 4:**
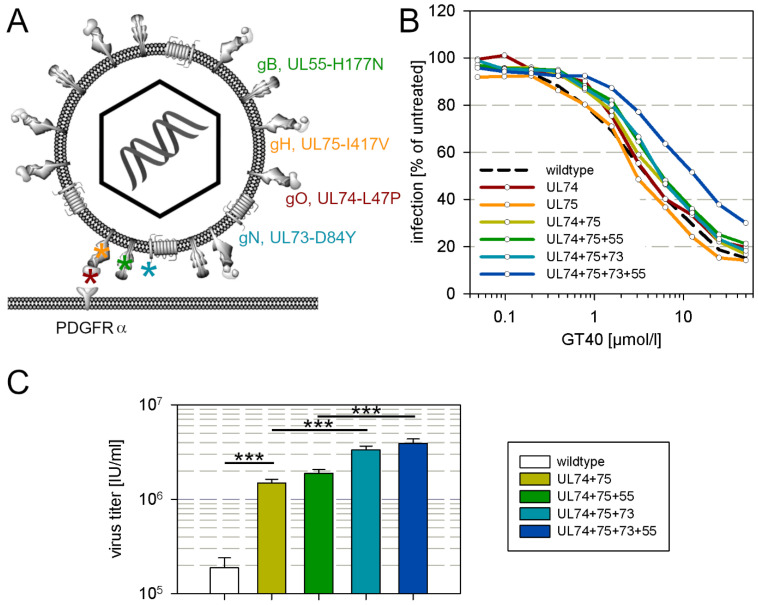
Mutations in envelope glycoproteins decrease sensitivity to GT40. (**A**) Localization of the mutations that were tested by marker transfer. (**B**) Each mutation or combination of mutations was introduced into two independent clones. Two independent virus supernatants were obtained from each clone. Thus, four virus supernatants were available for each combination, and each was independently tested twice, resulting in a total of eight replicates for each mutation combination. The supernatants were incubated for 2 h with different concentrations of GT40 and then added to HFFs. After overnight incubation, viral IE-Ag was stained by immunofluorescence and the number of infected cells compared to the untreated control was determined as the relative infection rate and plotted against the peptide concentration. (**C**) HFFs were infected at a low infection multiplicity of 0.01 infectious units per cell with wildtype pHB5 or mutants as indicated, GT40 was added at 25 µmol/L 3 d post infection and refreshed after 3 days. Virus supernatants were harvested at 11 and 12 d after infection, and the virus titers in the supernatants were determined. Bars indicate the mean of four experiments at 11 d and four experiments at 12 d; error bars indicate the standard error of the mean. Asterisks indicate statistical significance of the differences (*** = *p* < 0.001).

This clone was analyzed by whole genome sequencing for mutations as compared with the wildtype AD169 sequence, yielding a list of 105 mutations on the nucleotide level of which 65 occurred in known open reading frames. These mutations translate into 25 mutations on the amino acid level, that are dispersed over the whole genome. This large number of mutations was not unexpected due to the proofreading deficiency of the parental strain. Among these mutations however, four were found in envelope glycoprotein complexes I, II and III, and were therefore considered the most plausible candidates for a causal relation with the partial resistance against the entry inhibitor GT40, i.e., L47P in gO, I417V in gH, D84Y in gN and H177N in gB (Figure 4A).

To test whether these mutations confer a reduction in sensitivity to GT40, we performed marker transfer experiments by introducing them into the genetic backbone of the proofreading-intact pHB5 clone of HCMV strain AD169 and analyzed the reconstituted mutant viruses for sensitivity to GT40. As PDGFRα-Fc binds to the gH/gL/gO trimer in the viral envelope, we first introduced the mutations in gO (L47P) and gH (I417V) alone and in combination into pHB5, which only had a marginal effect on the sensitivity to GT40 as compared to wildtype virus (Figure 4B). Further addition of H177N in gB and D84Y in gN caused a further slight shift of the dose–response curve towards higher EC50 values, but only when all four mutations were combined did the reduction in sensitivity become clear, with a four-fold increase in the EC50 (Figure 4B), which resembled the reduction in sensitivity measured with the selected virus (Figure 3A) and the purified clone in which the mutations have been found (Figure 3B). To further corroborate these findings by a different assay, we compared the UL74 + 75 mutant, the UL74 + 75 + 55 mutant, the UL74 + 75 + 73 mutant and the complete UL74 + 75 + 73 + 55 mutant regarding their ability to grow and produce progeny in a multistep growth analysis. HFFs were infected with each mutant and wildtype virus at a low infection multiplicity of 0.01 infectious units per cell, resulting in 1% infected cells in each culture. GT40 was added at submaximal inhibitory concentrations (25 µmol/mL), virus supernatants were harvested at 11 and 12 d after infection, and the virus titers were compared. In this assay, already the UL74 + 75 mutation facilitated viral growth under these conditions by factor 10 and this was further enhanced by the UL73-mutation, whereas the UL55-mutation had only a slight, nonsignificant effect (Figure 4C).

## 4. Discussion

A soluble derivative of the cellular HCMV receptor PDGFRα, the chimeric molecule PDGFRα-Fc in which the complete extracellular domain of the receptor is fused to the Fc part of human immunoglobulin G, can inhibit viral entry and may represent a novel antiviral approach for treatment and prophylaxis of HCMV infections [9,12,19]. We hypothesized that such receptor-derived entry inhibitors have a high genetic barrier to resistance, as any viral mutation that reduces binding of the decoy receptor may also probably affect binding of the virus to this receptor on the cell surface. Our results support this notion, as only a mutant with moderately reduced sensitivity could be selected. The 40mer peptides GT40 and IK40, derived from the extracellular domain of PDGFRα, were also identified as inhibitors of HCMV, and our data suggest that they resemble the complete protein regarding an effect on adsorption and/or penetration of HCMV. Remarkably, a combination of selected mutations was necessary to confer at least a moderate reduction of sensitivity against GT40, and no resistance at all could be selected for IK40, indicating that the genetic barrier is also high for these entry inhibitors, which was less expected than for the complete molecule.

The serine to tyrosine mutation at position 48 in the N-terminus of gO that reduced sensitivity to PDGFRα-Fc by about three-fold is located close to a region of this protein that has previously been identified as relevant for receptor binding in a mutational scanning approach [20], providing further support for the functional relevance of the N-terminus. The structure of gO bound to PDGFRα has been resolved by cryo-electron microscopy, and these data indicate that the N-terminus of gO interacts with domain 2 of the receptor [21], which has been identified as relevant for inhibition of HCMV by multiple approaches [9,12,19,22]. As we did not find evidence for a reduced fitness of the mutant virus, i.e., virus titers did not differ from wildtype virus (Figure S1), it is tempting to speculate that subtle differences between the receptor on the cell surface and the soluble Fc-chimera exist that allow slightly reduced binding of the inhibitor while receptor-binding of the virus is unaffected. The fact that transfer of the S48Y mutation into gO of the AD169-BAC pHB5 did not exactly mediate the reduction of sensitivity as seen in the selected virus leaves the possibility of a second mutation in another protein, that might add to the effect of the gO-mutation. Taken together, this supports the assumption that the genetic barrier to the development of resistance to PDGFRα-Fc is high. Continued selection under increased selective pressure might reveal whether a more profound escape is possible by acquisition of additional mutations.

The results obtained with the peptides provide two pieces of information, one regarding the potential interaction sites and one regarding the barrier to resistance. The S48Y mutation that conferred a moderate level of resistance against PDGFRα-Fc sensitized the virus for inhibition by GT40, indicating that this part of gO is involved in the interaction of this peptide with the virus. In line with this assumption, selection with GT40 induced the mutation L47P in the neighboring amino acid, and recent structural data also show that GT40 is located in a loop of domain 2 within PDGFRα that includes three of the five hydrophobic residues in the designated interaction site 3 that are directed to a hydrophobic groove at the N terminus of gO [21]. IK40 locates directly at interaction site 4 within domain 3 of PDGFRα, which interacts with the C-terminal part of gO [21], and therefore it appears plausible that its inhibitory potential is not affected by a mutation in the N-terminus of gO. Remarkably, IK40 and GT40 also differed with respect to the step during virus entry that they prevent, with IK40 acting almost exclusively on attachment, whereas GT40 reduces both attachment and penetration, which closely resembles the effect of the complete PDGFRα-Fc molecule [9]. Surprisingly, our attempts to select resistant mutants suggest that the genetic barrier to resistance is even higher for PDGFRα-derived peptides than for the complete molecule. Our expectation was that it is easier for the virus to develop mutations against the peptides, as they certainly differ structurally from the receptor on the cell surface more than PDGFRα-Fc. Failure to induce a detectable loss of sensitivity with IK40 leaves the possibility that, other than expected, this peptide may primarily act on the cell rather than on the virus. The same applies, to a lesser extent, to GT40 as we found numerous mutations in the virus that developed under selection pressure, and it took a combination of mutations in gO (UL74) and gH (UL75) to confer at least a moderate loss of sensitivity, which was further increased by mutations in gN (UL73) and gB (UL55). We selected these four mutations for the marker transfer experiment as they were located in envelope glycoproteins that are presumably involved in attachment and penetration [11]. When searching for explanations as to why mutations in gB and gN were induced during selection with GT40, indirect compensatory effects also have to be considered, because both proteins are most probably not primarily involved in binding to PDGFRα [7,23,24]. Given that the mutations in gO and gH have a direct effect on binding of GT40, but also on binding to the receptor on the cell surface, it is conceivable that the mutation in gB compensates for a replication disadvantage of mutations in gH and gO by facilitating fusion activity. Concordant with this speculation, the mutation H177N is in an α-helix domain 1 that is located between the two fusion loops [25]. The finding that only a combination of mutations could reduce the sensitivity to GT40 leaves ambiguity regarding its molecular target but, on the other hand, it is encouraging regarding its therapeutic potential.

In conclusion, a single S48Y mutation in the N-terminus of gO conferred moderate resistance against PDGFRα-Fc, indicating that this domain contributes to the interaction between the viral glycoprotein complex gH/gL/gO and the cellular receptor PDGFRα. The fact that this mutation does not render the virus resistant against PDGFRα-derived peptides suggests that they differ from PDGFRα-Fc regarding their mode of action. Sequence analysis of the virus selected with the PDGFRα-derived peptide GT40 and initial marker transfer data indicate that a complex combination of mutations in all three envelope glycoprotein complexes is necessary to reduce its inhibitory effect, suggesting a high genetic barrier to the development of resistance.

## Figures and Tables

**Table 1:** Primers used for the introduction of candidate resistance-conferring mutations into the genome of HCMV pHB5.

Primer	Sequence (5′-3′)
UL74-S48Y for	TTCTAACTTAAGCTGATCTAATTTATATTTGCCTATCTTATACAGTACCAAGCCCCTCCAAaggatgacgacgataagt
UL74-S48Y rev	AAAGCGCTTTATAATCGTCCTTGGAGGGGCTTGGTACTGTATAAGATAGGCAAATATAAATcaaccaattaaccaattctga
UL74-S48Y short for	TTCTAACTTAAGCTGATCTA
UL55-H177N for	CTGTAGGAACTGTAGCATTGAGCAAACTTGTTGATGTGATTAATCTCCCACATAGGAGGCGaggatgacgacgataagt
UL55-H177N rev	CAGCAATACGGAATACGTGGCGCCTCCTATGTGGGAGATTAATCACATCAACAAGTTTGCTcaaccaattaaccaattctga
UL55-H177N short for	CTGTAGGAACTGTAGCATTG
UL73-D84Y for	CACCCACGATCCTAATGTGATGAGACGACATGCGAACGATTATTTTTACAAGGCGCATTGCaggatgacgacgataagt
UL73-D84Y rev	AGCTCATACATATGCGATGTGCAATGCGCCTTGTAAAAATAATCGTTCGCATGTCGTCTCAcaaccaattaaccaattctga
UL73-D84Y short for	CACCCACGATCCTAATGTGA
UL74-L47P for	TAACTTAAGCTGATCTAATTTATATTTGCCTATCTTAGACGGTACCAAGCCCCTCCAAGGAaggatgacgacgataagt
UL74-L47P rev	TCAAAAGCGCTTTATAATCGTCCTTGGAGGGGCTTGGTACCGTCTAAGATAGGCAAATATAcaaccaattaaccaattctga
UL74-L47P short for	TAACTTAAGCTGATCTAATT
UL75-I417V for	TGTTTAGAAAGTATGTAGACCAGGCGTACGAGGCTGGTGACGTCGGTGATCTGGTCCGGCGaggatgacgacgataagt
UL75-I417V rev	GGCCAAACGAGCCCTCTGGACGCCGGACCAGATCACCGACGTCACCAGCCTCGTACGCCTGcaaccaattaaccaattctga
UL75-I417V for short	TGTTTAGAAAGTATGTAGAC
Kanamycin universal reverse	CAACCAATTAACCAATTCTGA

Note: primer sites encoding mutated codons are underlined; uppercase letters: HCMV sequence; lowercase letters: Kanamycin cassette.

**Table 2:** Primers used for generation of the dual fluorescent virus TB40-BAC-KL7-SE-UL32EGFP-UL100mCherry.

Primer	Sequence (5′-3′)
EGFP for	CCGTGCAGAACATCCTCCAAAAGATCGAGAAGATTAAGAAAACGGAGGAAatggtgagcaagggcgaggagct
EGFP rev	CACTATCCGATGATTTCATTAAAAAGTACGTCTGCGTGTGTGTTTCTTAAttacttgtacagctcgtccatgc
mCherry for	CCCTGCGTCTACTATCACGTCGTGGACTTTGAAAGGCTCAACATGTCGGCCTACAACGTAgtgagcaagggcgaggagga
mCherry rev	CACGGCGTAGCACACCAGCTGCACCGAGTCTAAGAAAAGCATAGGCGTGTGCAGGTGCATcttgtacagctcgtccatgc

Note: uppercase letters: HCMV sequence; lowercase letters: template plasmid (EGFP or mCherry sequence).

## Data Availability

The complete sequence of clone 1 (HGUL54dD413 selGT40 clone1) is available at the NCBI GenBank with accession number MZ327301.

## Supplementary Materials

The following are available online at https://www.mdpi.com/article/10.3390/v13061094/s1, Figure S1: Human fibroblast cultures (HFFs) were infected with the respective virus, and cell-free supernatant was harvested when the late-stage cytopathic effect had fully developed. The supernatants were then analyzed for virus titers. Indicator HFFs on 96-well plates were infected with serial dilutions of each supernatant, incubated overnight, fixed with 80 % acetone, and stained for viral immediate early antigen by indirect immunofluorescence, yielding red nuclear fluorescence signals. Cell nuclei were subsequently counterstained with DAPI, resulting in blue nuclear fluorescence. The fraction of infected cells was counted, the half-maximal concentration (EC50) was determined by nonlinear regression. The corresponding virus titers were calculated from the EC50 values, and the mean values for each virus were determined and presented as bars. The error bars represent the standard error of the mean (UL74-S48Y, n = 3; wildtype, n = 3; UL74 + 75 + 73 + 55, n = 4). Differences between mutant viruses and wild-type virus were not significant (n.s.).

## References

[1] Razonable R.R. (2018). Drug-resistant cytomegalovirus: Clinical implications of specific mutations. Curr. Opin. Organ Transplant..

[2] Marty F.M., Ljungman P., Chemaly R.F., Maertens J., Dadwal S.S., Duarte R.F., Haider S., Ullmann A.J., Katayama Y., Brown J. (2017). Letermovir Prophylaxis for Cytomegalovirus in Hematopoietic-Cell Transplantation. N. Engl. J. Med..

[3] Lischka P., Michel D., Zimmermann H. (2016). Characterization of Cytomegalovirus Breakthrough Events in a Phase 2 Prophylaxis Trial of Letermovir (AIC246, MK 8228). J. Infect. Dis..

[4] Gulick R.M. (2018). Investigational Antiretroviral Drugs: What is Coming Down the Pipeline. Top. Antivir. Med..

[5] Haqqani A.A., Tilton J.C. (2013). Entry inhibitors and their use in the treatment of HIV-1 infection. Antivir. Res..

[6] Bogomolov P., Alexandrov A., Voronkova N., Macievich M., Kokina K., Petrachenkova M., Lehr T., Lempp F.A., Wedemeyer H., Haag M. (2016). Treatment of chronic hepatitis D with the entry inhibitor myrcludex B: First results of a phase Ib/IIa study. J. Hepatol..

[7] Wu Y., Prager A., Boos S., Resch M., Brizic I., Mach M., Wildner S., Scrivano L., Adler B. (2017). Human cytomegalovirus glycoprotein complex gH/gL/gO uses PDGFR-α as a key for entry. PLoS Pathog..

[8] Kabanova A., Marcandalli J., Zhou T., Bianchi S., Baxa U., Tsybovsky Y., Lilleri D., Silacci-Fregni C., Foglierini M., Fernandez-Rodriguez B.M. (2016). Platelet-derived growth factor-α receptor is the cellular receptor for human cytomegalovirus gHgLgO trimer. Nat. Microbiol..

[9] Stegmann C., Hochdorfer D., Lieber D., Subramanian N., Stöhr D., Laib Sampaio K., Sinzger C. (2017). A derivative of platelet-derived growth factor receptor alpha binds to the trimer of human cytomegalovirus and inhibits entry into fibroblasts and endothelial cells. PLoS Pathog..

[10] Martinez-Martin N., Marcandalli J., Huang C.S., Arthur C.P., Perotti M., Foglierini M., Ho H., Dosey A.M., Shriver S., Payandeh J. (2018). An Unbiased Screen for Human Cytomegalovirus Identifies Neuropilin-2 as a Central Viral Receptor. Cell.

[11] Nguyen C.C., Kamil J.P. (2018). Pathogen at the Gates: Human Cytomegalovirus Entry and Cell Tropism. Viruses.

[12] Feldmann S., Grimm I., Stöhr D., Antonini C., Lischka P., Sinzger C., Stegmann C. (2021). Targeted mutagenesis on PDGFRα-Fc identifies amino acid modifications that allow efficient inhibition of HCMV infection while abolishing PDGF sequestration. PLoS Pathog..

[13] Goldner T., Hewlett G., Ettischer N., Ruebsamen-Schaeff H., Zimmermann H., Lischka P. (2011). The novel anticytomegalovirus compound AIC246 (Letermovir) inhibits human cytomegalovirus replication through a specific antiviral mechanism that involves the viral terminase. J. Virol..

[14] Borst E.M., Hahn G., Koszinowski U.H., Messerle M. (1999). Cloning of the human cytomegalovirus (HCMV) genome as an infectious bacterial artificial chromosome in Escherichia coli: A new approach for construction of HCMV mutants. J. Virol..

[15] Tischer B.K., Smith G.A., Osterrieder N. (2010). En passant mutagenesis: A two step markerless red recombination system. Methods Mol. Biol. Clifton NJ.

[16] Laib Sampaio K., Weyell A., Subramanian N., Wu Z., Sinzger C. (2017). A TB40/E-derived human cytomegalovirus genome with an intact US-gene region and a self-excisable BAC cassette for immunological research. BioTechniques.

[17] Jiang X.J., Sampaio K.L., Ettischer N., Stierhof Y.-D., Jahn G., Kropff B., Mach M., Sinzger C. (2011). UL74 of human cytomegalovirus reduces the inhibitory effect of gH-specific and gB-specific antibodies. Arch. Virol..

[18] Hirt B. (1967). Selective extraction of polyoma DNA from infected mouse cell cultures. J. Mol. Biol..

[19] Park J., Gill K.S., Aghajani A.A., Heredia J.D., Choi H., Oberstein A., Procko E. (2020). Engineered receptors for human cytomegalovirus that are orthogonal to normal human biology. PLoS Pathog..

[20] Stegmann C., Rothemund F., Laib Sampaio K., Adler B., Sinzger C. (2019). The N Terminus of Human Cytomegalovirus Glycoprotein O Is Important for Binding to the Cellular Receptor PDGFRα. J. Virol..

[21] Kschonsak M., Rougé L., Arthur C.P., Hoangdung H., Patel N., Kim I., Johnson M.C., Kraft E., Rohou A.L., Gill A. (2021). Structures of HCMV Trimer reveal the basis for receptor recognition and cell entry. Cell.

[22] Wu K., Oberstein A., Wang W., Shenk T. (2018). Role of PDGF receptor-α during human cytomegalovirus entry into fibroblasts. Proc. Natl. Acad. Sci. USA.

[23] Wille P.T., Wisner T.W., Ryckman B., Johnson D.C. (2013). Human cytomegalovirus (HCMV) glycoprotein gB promotes virus entry in trans acting as the viral fusion protein rather than as a receptor-binding protein. MBio.

[24] Wussow F., Chiuppesi F., Contreras H., Diamond D.J. (2017). Neutralization of Human Cytomegalovirus Entry into Fibroblasts and Epithelial Cells. Vaccines.

[25] Liu Y., Heim K.P., Che Y., Chi X., Qiu X., Han S., Dormitzer P.R., Yang X. (2021). Prefusion structure of human cytomegalovirus glycoprotein B and structural basis for membrane fusion. Sci. Adv..

