# Supplementary material for: Selection of Human Cytomegalovirus Mutants with Resistance against PDGFRα-Derived Entry Inhibitors
Source: Viruses. 2021 Jun 8;13(6):1094. doi: 10.3390/v13061094 (PMC8229732; doi:10.3390/v13061094)
Supplement: Supplementary file 1 [file viruses-13-01094-s001.zip › viruses-1224490-supplementary.pdf]

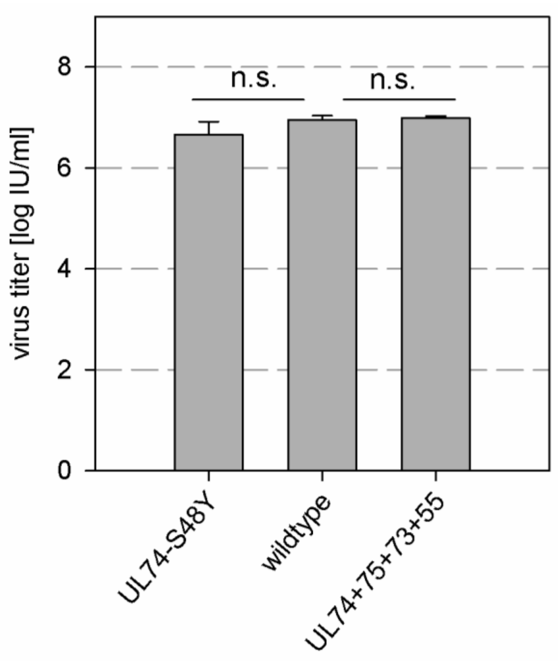

Figure S1: Human fibroblast cultures (HFFs) were infected with the respective virus, and cell-free supernatant was harvested when the late-stage cytopathic effect had fully developed. The supernatants were then analyzed for virus titers. Indicator HFFs on 96-well plates were infected with serial dilutions of each supernatant, incubated overnight, fixed with 80 % acetone, and stained for viral immediate early antigen by indirect immunofluorescence, yielding red nuclear fluorescence signals. Cell nuclei were subsequently counterstained with DAPI, resulting in blue nuclear fluorescence. The fraction of infected cells was counted, the half-maximal concentration (EC50) was determined by nonlinear regression. The corresponding virus titers were calculated from the EC50 values, and the mean values for each virus were determined and presented as bars. The error bars represent the standard error of the mean (UL74-S48Y, n=3; wildtype, n=3; UL74+75+73+55, n=4). Differences between mutant viruses and wild-type virus were not significant (n.s.).
